# Application of Fluorescence-Based Probes for the Determination of Superoxide in Water Treated with Air Non-thermal Plasma

**DOI:** 10.1021/acssensors.0c01042

**Published:** 2020-08-17

**Authors:** Gabriele Cabrellon, Francesco Tampieri, Andrea Rossa, Antonio Barbon, Ester Marotta, Cristina Paradisi

**Affiliations:** Department of Chemical Sciences, University of Padova, Via Marzolo 1, 35131 Padova, Italy

**Keywords:** cold plasma, superoxide steady-state concentration, superoxide lifetime, superoxide production rate, superoxide probe, spin-trapping

## Abstract

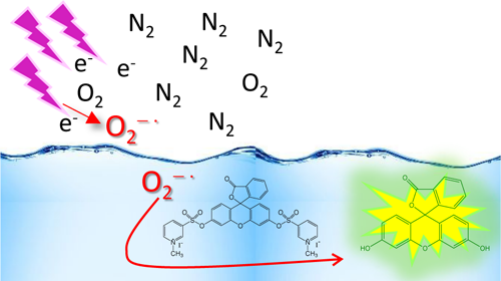

Superoxide is one of the reactive oxygen species (ROS) in non-thermal plasmas generated by electrical discharges in air at room temperature and atmospheric pressure. One important application of such plasmas is the activation of advanced oxidation processes for air and water decontaminating treatments. When in contact with aqueous media, ROS and notably superoxide can react at the plasma/liquid interface or transfer and react into the liquid. While the detection of superoxide in plasma-treated water has been reported in the literature, to the best of our knowledge, quantitative determinations are lacking. We report here the determination of superoxide rate of formation and steady-state concentration in water subjected to air non-thermal plasma in a streamer discharge reactor used previously to treat various organic contaminants. After detecting the presence of superoxide by spin-trapping and electron paramagnetic resonance analyses, we applied superoxide-selective fluorescent probes to carry out quantitative determinations. The first probe tested, 3′,6′-bis(diphenylphosphinyl) fluorescein (PF-1), was not sufficiently soluble, but the second one, fluorescein-bis-[(*N*-methylpyridinium-3-yl)sulfonate iodide] (FMSI), was applied successfully. Under typical plasma operating conditions, the rate of superoxide formation and its steady-state concentration were (0.27 ± 0.15) μM s^–1^ and (0.007 ± 0.004) nM, respectively. The procedure outlined here can be usefully applied to detect and quantify superoxide in water treated by different plasma sources in various types of plasma reactors.

Air non-thermal plasma in contact with water creates a very complex heterogeneous system (gas/plasma/liquid) comprising a cocktail of reactive species including reactive oxygen species (ROS), reactive nitrogen species (RNS), electrons, and photons.^1^ Depending on the specific target of the plasma treatment, however, not all such species are equally useful. Thus, for example, since OH radicals are generally very efficient initiators for reaction of organic compounds in advanced oxidation processes in water treatment, plasma sources, reactor design, and experimental conditions are usually designed in such a way as to maximize OH radical production in solution. There are, however, exceptions, notably perfluoroalkyl substances which do not react with OH radicals but are most efficiently treated by electron-rich plasmas.^2^ It is therefore not surprising that much current research is focused on determining all major plasma-induced reactive species, the mechanisms of their production and transformation, and their transport and partitioning among phases. The behavior of reactive species is usefully discussed in the framework of reactivity/selectivity correlations. Thus, among ROS, ozone is relatively stable, at least in acidic/neutral solutions, and selective in its reactions and can be determined by direct measurements. The same holds for hydrogen peroxide. In contrast, as mentioned above, OH radicals are very reactive and are detected and determined by indirect methods based on trapping by suitable molecular probes to produce either stable radicals which can be analyzed by electron paramagnetic resonance (EPR) spectroscopy (e.g., 5,5-dimethyl-1-pyrroline *N*-oxide (DMPO) forms a radical adduct that is more stable than the parent radical)^3^ or fluorescent products, which can be quantified by fluorimetric determinations (e.g., coumarin-3-carboxylic acid (CCA), giving the fluorescent product 7-hydroxycoumarin-3-carboxylic acid (7-OH-CCA),^4,5^ or terephthalate (TPA), giving the fluorescent product 2-hydroxyterephtalate (hTPA)).^6^ With a lifetime in water of the order of microseconds, the radical ion superoxide, O_2_^–•^, is less reactive than the OH radical.^7,8^ In the gas phase, superoxide is readily observed directly in atmospheric pressure mass spectrometry (APCI-MS) analysis. In previous work, we found that O_2_^–•^ and its hydrated clusters O_2_^–•^(H_2_O)_*n*_ are the major ionic species in dc- corona discharges in air.^9,10^

In the solid state, superoxide is stable as potassium salt and can be stored for years under anhydrous conditions.^11^ In aqueous media, the main reactions of superoxide are disproportionation, proton abstraction (the p*K*_a_ of ^•^OOH is 4.8), one-electron transfer, and nucleophilic substitution.^11^ When KO_2_ is added to water, a vigorous reaction occurs, forming oxygen, hydroxide, and hydroperoxide (eq 1), followed by slower decay of hydroperoxide to hydroxide (eq 2).^11,12^ KO_2_ is commonly used as a source of superoxide to prepare relatively stable solutions in polar aprotic solvents such as dimethylsulfoxide (DMSO).
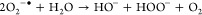
1
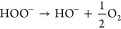
2

Detection and quantification of superoxide is of paramount importance in biomedical research, and many assays and probes based on the release of fluorescent products^13^ have been studied and developed into commercial kits.^14^ In plasma/liquid research, the presence of superoxide in the aqueous phase has been inferred by EPR spectroscopy using suitable spin traps.^3,8,15−19^ In most of these studies, DMPO has been employed, which reacts with superoxide to form a relatively stable adduct radical, DMPO–OOH, with characteristic spectral properties.^20^ There are, however, limitations which hinder the possibility to obtain quantitative data by this approach. First, DMPO also reacts with other radicals, including the OH radical. Second, the DMPO–OOH adduct is not very stable, with a lifetime of about 1 min, and evolves to form DMPO–OH, the DMPO adduct with ^•^OH; thus, the signals of DMPO–OH can be due to the trapping of both ^•^OH and ^•^OOH. Tani et al. detected the DMPO–OOH adduct in aqueous media treated with different He plasma sources in the presence of oxygen.^21^ The authors showed that the adduct was not observed if superoxide dismutase (SOD), a quencher of superoxide, was also present in solution. Wu et al.^17^ detected superoxide indirectly by showing that the signal intensity of DMPO–OH is reduced by addition of SOD and speculated that superoxide is one of the main precursors of OH radicals. Other spin traps have also been tested to detect and study plasma-generated superoxide in solutions, such as 5-(2,2-dimethyl-1,3-propoxycyclophosphoryl)-5-methyl-1-pyrroline-*N*-oxide) (CYMPO), 5-(diethoxyphosphoryl)-5-methyl-1-pyrroline *N*-oxide (DEPMPO), and 5-*tert*-butoxycarbonyl-5-methyl-1-pyrroline-*N*-oxide (BMPO).^3,8,15,21,22^ These have the advantage that their adducts with superoxide are more stable than that with DMPO and do not evolve to the corresponding adducts with the hydroxyl radical. However, the widespread use of these spin traps in many studies was prevented by their high costs and relatively low availability.

By reviewing the literature in search for previous attempts to quantify plasma-produced superoxide in solution, we found a few papers describing sound work and interesting results but reporting misleading assignment of the measured quantities to superoxide concentration. For example, Tresp et al.^8^ have used both DMPO and BMPO to investigate the formation of superoxide and hydroxyl radicals in phosphate-buffered saline solution treated with an atmospheric pressure argon plasma jet with varying mixtures of oxygen and nitrogen as shielding gas. The authors determined the rate of formation and concentration of the spin-trap adducts obtained by quantitative EPR measurements, which turned out to be in the 1–5 μM range, and compared these values with the concentrations of ROS free radicals normally found in biological systems (<0.1 pM). Similarly, spin-trapping experiments were carried out with DMPO to detect and quantify OH and superoxide radicals in physiological solutions and cell culture media subjected to non-thermal plasma.^18^ The concentrations of ROS in these systems were incorrectly assumed to be equal to the measured concentrations of their spin-trap adducts, which again were in the micromolar range. Similar conclusions are presented in a paper by Jose et al.,^23^ with reported concentrations of superoxide between 0.010 and 0.050 mM, whereas these data refer to the amounts of trapped superoxide and not to its instantaneous concentration. Therefore, any comparison of these values with those found in biological systems, which are in the picomolar range, is meaningless. Specifically, the measured instantaneous concentration of any adduct between ROS and a spin trap cannot be considered equal to the steady-state concentration of the ROS itself but is much higher because the lifetime of ROS-spin-trap adducts is much longer than that of free ROS. Thus, ROS-spin-trap adducts accumulate in time in the plasma-treated solution. This is true even considering that only a fraction of all produced ROS is being captured by the trap and that the trap itself can be consumed by reactions besides that with the monitored ROS.

In conclusion, strong evidence for the production of superoxide in water treated with non-thermal plasma is available based on EPR methods, but, so far, no reliable quantitative data of superoxide rates of formation and steady-state concentrations in these systems have been reported. These data can be accessed using proper kinetic modelling to take into account all processes mentioned above. One such model was proposed by Anifowose et al. and applied to determine superoxide in natural sea waters exposed to sunlight.^24^

The purpose of the research reported in this paper was the unambiguous identification of superoxide generated in water treated with air non-thermal plasma and its quantitative determination (Scheme 1). We used a streamer discharge plasma reactor developed earlier in our laboratory and applied successfully to treat water contaminated with organic pollutants.^25^ In these earlier investigations, various other reactive species had been detected and determined, including the OH radical, hydrogen peroxide, and ozone but not superoxide. To tackle this task, we first sought and obtained qualitative evidence for the presence of superoxide by means of spin-trapping experiments, using DEPMPO as the spin trap, and EPR analysis. Next, we adapted to our system a test based on suitable probes, which upon selective trapping of superoxide release fluorescein (FL) that can be readily quantified by highly sensitive fluorimetric measurements. The first probe we tried was 3′,6′-bis(diphenylphosphinyl) fluorescein, (PF-1).^26^ Inspired by the work of Anifowose et al., who used PF-1 to determine the rate of photoinduced formation of superoxide and its steady-state concentration in surface ocean waters,^24^ we synthesized PF-1 and tested it in our plasma reactor. Although PF-1 gave us evidence for the presence of superoxide in plasma-treated water, it was not possible to carry out quantitative determinations because of the insufficient solubility of the probe. We thus turned to a different probe: fluorescein-bis-[(*N*-methylpyridinium-3-yl)sulfonate iodide] (FMSI), a new FL derivative, recently developed and described in the literature as highly soluble in water and highly selective toward superoxide with no interference by other reactive species.^27^ With FMSI, we indeed succeeded in performing quantitative measurements and, following the procedure of Anifowose et al.,^24^ obtained values for the rate of formation and steady-state concentration of superoxide in water treated in our streamer discharge reactor.

**Scheme 1:**
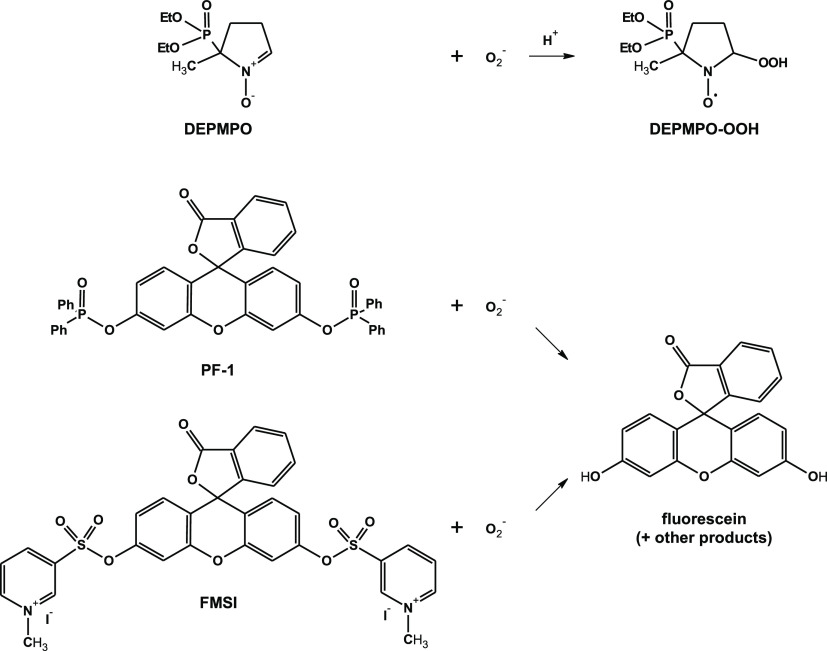
Structures of DEPMPO, PF-1, and FMSI and Their Reactions with Superoxide

## Experimental Section

### Materials

Fluorescein (FL) was purchased from TCI chemicals; *N*,*N*-diisopropylethylamine (≥99%), triethylamine (TEA, ≥99.5%), iodomethane (99%), pyridine-3-sulfonyl chloride (≥99%), diphenylphosphinic chloride (Ph_2_POCl 98%), hydrogen peroxide (H_2_O_2_, 30%), anhydrous tetrahydrofuran (THF, ≥99.9%), anhydrous dimethyl sulfoxide (DMSO, ≥99.9%), *N*,*N*-dimethylformamide (DMF, HPLC grade), deuterated dimethyl sulfoxide (DMSO-*d*_6_, 99.9%), deuterated chloroform (CDCl_3_, ≥99%), potassium dioxide (KO_2_), trifluoroacetic acid (≥99%), and formic acid (≥98%) were purchased from Sigma-Aldrich; 5-(diethoxyphosphoryl)-5-methyl-1-pyrroline *N*-oxide (DEPMPO, 99%) was purchased from Focus Biomolecules; fluoranil was purchased from EGA-Chemie (97%); iron (II) ammonium sulfate hexahydrate (Fe(NH_4_)_2_(SO_4_)_2_, ≥99.0%) was purchased from Fluka; methanol (HPLC grade) was purchased from VWR; potassium dihydrogen phosphate (KH_2_PO_4_, ≥99%), disodium hydrogen phosphate dodecahydrate (Na_2_HPO_4_·12 H_2_O, ≥ 99%), and acetonitrile (HPLC grade) were purchased from Carlo Erba; petroleum ether, ethyl acetate, and dichloromethane (DCM) were purchased from Honeywell Riedel-de Haën. Ultrapure grade water (MilliQ water) was obtained by filtration of deionized water with a Millipore system. “Synthetic air” (a N_2_/O_2_ 80:20 mixture) was purchased from Air Liquide. All reagents and solvents were used as received without further purification, unless otherwise specified.

### Synthesis of Probes

#### PF-1

The synthesis of PF-1 was performed by adapting the procedure of Xu et al.^28^ TEA (1.20 mL, 8.61 mmol, 2.9 equiv) and Ph_2_POCl (1.73 mL, 9.07 mmol, 3.0 equiv) were added to a solution of FL (1.00 g, 3.01 mmol, 1.0 equiv) in anhydrous THF (45 mL), and the mixture was stirred for 2 h at 70 °C. The precipitated triethylamine hydrochloride was separated by filtration, and the solvent of the resulting solution was removed under reduced pressure. The residue was dissolved in DCM (100 mL) and washed with 0.5 M HCl (200 mL). The aqueous layer was extracted with DCM (2 × 100 mL), the combined organic layers were dried over Na_2_SO_4_, and the solvent was removed under reduced pressure. The residue was purified by silica gel flash chromatography (first column EtOAc/PE 7.5:2.5; second column DCM/EtOAc 7:3) to afford PF-1 (1.50 g, 2.05 mmol, 68% yield) as a white solid. The final product was characterized by nuclear magnetic resonance (NMR) and MS analyses. NMR spectra were recorded with a Bruker AVII500 UltraShield spectrometer operating at 500 MHz (for ^1^H NMR) and 126 MHz (for ^13^C NMR) in a solution of deuterated chloroform (CDCl_3_). Chemical shifts (δ) are given in parts per million (ppm) relative to the signal of residual CHCl_3_ (δ 7.26 ppm for ^1^H NMR, δ 77.16 ppm for ^13^C NMR). The following abbreviations are used to indicate multiplicities: d, doublet; dd, doublet-of-doublets; ddd, doublet-of-doublets-of-doublets; m, multiplet. MS analysis was performed by an Agilent Technologies MSD SL Trap system equipped with an electrospray source and an ion trap analyzer. ^1^H NMR (500 MHz, CDCl_3_): δ 7.98–7.94 (m, 1H), 7.91–7.83 (m, 8H), 7.64–7.51 (m, 6H), 7.50–7.43 (m, 8H), 7.14 (dd, *J* = 2.3, 1.1 Hz, 2H), 7.07–7.03 (m, 1H), 6.88 (ddd, *J* = 8.8, 2.4, 1.0 Hz, 2H), 6.63 (d, *J* = 8.7 Hz, 2H). ^13^C NMR (126 MHz, CDCl_3_): δ 169.2, 152.8, 152.4 (d, *J* = 8.1 Hz), 151.8, 135.3, 132.9 (m), 131.8 (dd, *J* = 10.4, 1.9 Hz), 130.6 (dd, *J* = 138.0, 6.9 Hz), 130.1, 129.4, 128.9 (dd, *J* = 13.6, 3.1 Hz), 126.3, 125.2, 124.1, 116.9 (d, *J* = 4.9 Hz), 115.3, 109.3 (d, *J* = 5.2 Hz), 81.9. ESI-MS: 733.2 *m*/*z* [M + H]^+^, 755.2 *m*/*z* [M + Na]^+^.

#### FMSI

The synthesis of FMSI was performed following the procedure of Lu et al.,^27^ slightly modified as described in our previous paper.^12^ NMR and MS data for the obtained product were the same as reported earlier.^12^

### Plasma Reactor

The experimental setup employed in this work is described in detail in a previous publication.^25^ Briefly, the reactor is a Pyrex cylindrical vessel (inner diameter (ID) = 4.1 cm, outer diameter (OD) = 4.5 cm, h = 6 cm, and volume 80 mL approx.) closed by a Teflon cover. The active electrode is a stainless steel tube (ID = 4.0 mm, OD = 6.0 mm) fixed through the cover and aligned with the cylinder axis, which also serves as the inlet port for the plasma feed gas (“synthetic air” in the present work). The active electrode ends in a flared tip, with an ID of 0.5 mm. The final portion of the active electrode is embedded in a Pyrex tube, protruding beyond the electrode tip and dipped inside the solution to be treated by about 6 mm. Through the tip of the active electrode, which is in contact with the liquid, gas bubbles are released in the solution. A copper foil in contact with the external surface of the bottom of the vessel serves as the ground electrode.

The electrical excitation is provided by a high-voltage electronic transformer (*V*_p_ = 16 kV) that produces a modulated output in the 12–18 kHz frequency range. The average power delivered by the plasma is (5.9 ± 0.7) W.

During the experiments, “synthetic air” is flown at 100 mL min^–1^ through the active electrode and bubbled into the solution. To minimize evaporation from the solution, the air was presaturated with humidity by passing it through a water bubbler placed before the reactor. The volume of liquid used in the plasma treatment experiments was 15 mL. Kinetic studies were performed using a batch procedure, meaning that for each treatment time, a fresh experiment was carried out.

### Experimental Procedures

#### Spin-Trapping Experiments

A 9.33 mM solution of DEPMPO, prepared by dissolving pure DEPMPO in MilliQ water, was treated in the plasma reactor for 5 min. The solution was prepared and used within a few hours. In order to limit the consumption of this expensive spin-trapping reagent, a small volume (3.5 mL) of DEPMPO solution was treated in these experiments in a small glass cylindrical vessel (ID = 2.0 cm, OD = 2.4 cm) placed at the center of the plasma reactor. The depth of the DEPMPO solution inside the small vessel was the same as that of water surrounding it in the main reactor chamber. At the end of the plasma treatment, two small aliquots of solution were withdrawn from the reactor, transferred into small vials, and rapidly frozen in liquid nitrogen. Less than a minute lapsed between the switching off of the plasma discharge and the freezing of the treated solution. The same procedure was also used for aliquots of untreated DEPMPO solution to be used as the control. Untreated and plasma-treated solutions were then analyzed by EPR spectroscopy to detect radical species and by HPLC/ultraviolet−visible (UV−vis) to quantify unreacted DEPMPO.

For EPR analysis, the samples were thawed and N_2_ was bubbled in the solution for some seconds to remove oxygen. The solution was then transferred into an EPR flat cell (500 μL capacity) and subjected to EPR analysis. The time elapsed between defrosting of the solutions and spectra acquisition was about 5 min. Spectra were acquired at room temperature using an X-band Bruker ELEXSYS spectrometer equipped with a ER 4103TM cylindrical mode resonator for aqueous and high dielectric samples. The acquisition parameters were as follows: modulation frequency 100 kHz, scan range 150 G, modulation amplitude 1.8 G, receiver gain 60 dB, microwave frequency 9.78 GHz (scaling of the field was used), power attenuation 10 dB, time constant 40.96 ms, conversion time 81.92 ms, scan time 83.89 s, and number of scans 2. The EPR spectra were reproduced using Easyspin software^29^ in order to isolate and identify all radical species.

HPLC analyses were carried out with an Agilent 1260 Infinity II instrument (G7112B Binary Pump, G7129A Autosampler, G7114A VWD detector) on an Agilent InfinityLAb Poroshell 120 EC-C18 (2.7 μm, 3.0 × 150 mm) column using a mobile phase composed of MilliQ water with 0.1% formic acid (A) and CH_3_CN with 0.1% formic acid (B). The following gradient was used: from 0 to 3 min 5% B isocratic, from 3 to 6 min linear increase of B from 5 to 50%, and from 6 to 6.5 min 50% B isocratic; initial conditions were reestablished in 0.5 min. The flow rate was 0.4 mL/min.

#### Plasma Treatment of PF-1

To dissolve PF-1 in water, we used a procedure similar to that reported by Anifowose et al.^24^ Because of the low solubility of the probe in water, a 2 mM stock solution of PF-1 in DMF was diluted with aqueous phosphate buffer (5 mM, pH 7) to obtain a 35 μM final concentration. After plasma treatment of this solution, the samples were analyzed to quantify the amount of produced FL and unreacted PF-1. FL was quantified using a PerkinElmer LS-55B spectrofluorimeter and a quartz cuvette with an optical path of 10.0 mm. Spectra were recorded shortly after the treatment using the following parameters: λ_ex_ = 492 nm, range 500–600 nm, sampling rate 100 nm/min, and *T* = 25 °C. A calibration line was obtained by recording the fluorescence signal of standard solutions of FL prepared in the same phosphate buffer solution used for the plasma experiments. The concentration of residual PF-1 in solution was obtained by HPLC/UV−vis analysis. Instrument, column, and eluents used were the same as indicated above. The LC gradient for these analyses was from 0 to 1.5 min 30% B isocratic, from 1.5 to 8.5 min linear increase of B from 30 to 100%, and from 8.5 to 10 min 100% B isocratic; initial conditions were reestablished in 3 min. The flow rate was 0.7 mL/min. Elution of PF-1 was detected at 272 nm. Under the same conditions, FL is also detectable at 490 nm.

#### Plasma Treatment of FMSI

A 1 mM stock solution of FMSI was prepared in ultrapure water, slightly acidified to prevent decomposition.^12^ The working solutions were prepared directly in the reactor vessel immediately before the treatment by diluting the stock solution with the appropriate amount of phosphate buffer (200 mM, pH 7) to obtain the desired concentration. We have shown previously that at pH 7, FMSI is sufficiently stable in the experimental time scale.^12^ After plasma treatment, the samples were analyzed by ultrafast liquid chromatography (UFLC) to quantify the amount of produced FL and the unreacted probe. Emission and absorption UFLC chromatograms were recorded with a Shimadzu UFLC-XR instrument equipped with a Phenomenex Kinetex column (5 μm EVO C-18 100Å, 150 mm length and 4.6 mm internal diameter), an SPD-M20A diode array detector, and an RF-20A XS fluorescence detector. FMSI was detected by absorption at 190 nm, while FL was detected using a fluorescence detector (λ_ex/em_ = 492/513 nm). Retention times were 4.0 min for FMSI and 5.6 min for FL. Eluent was composed of H_2_O + 0.1% HCOOH (A) and CH_3_CN 0.1% HCOOH (B) with the following gradient: from 0 to 1.5 min 10% B isocratic, from 1.5 to 10 min linear increase of B from 10 to 60%, and from 10 to 12 min 60% B isocratic; initial conditions were reestablished in 3 min. The flow rate was 0.6 mL/min.

For some experiments, FL quantification was also performed by spectrofluorimetry using the conditions indicated in the previous section.

### Data Elaboration

To determine the rate of superoxide formation, *R*_O_2_^–^_ (eq 3), its lifetime, *t*_1/2_ (eq 4), and its steady state concentration, [O_2_^–^]_SS_ (eq 5), during our plasma treatment, we followed the procedure reported by Anifowose et al.,^24^ which is briefly outlined here for the readers convenience.
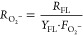
3
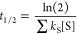
4
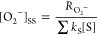
5

In these equations, *R*_FL_ is the rate of formation of FL, which was determined by fluorescence measurements as described above; *Y*_FL_ is the yield of FL formed by reaction of the probe with plasma-generated superoxide; *F*_O_2_^–^_ is the fraction of superoxide that reacts with the probe during the experiment; and ∑ *k*_S_[S] accounts for consumption of superoxide via its reactions with all other scavengers *S* present in the system (excluding the probe), each reacting with its specific rate constant *k*_S_. The yield of FL, *Y*_FL_, and the fraction of O_2_^–•^ that reacts with the probe, *F*_O_2_^–^_, were determined using eqs 6 and 7
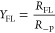
6
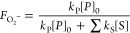
7where *R*_–P_ is the decay rate of the probe subjected to plasma treatment, which was determined by HPLC/UV–vis quantitative analyses as described above, *k*_P_ is the rate constant for the reaction of superoxide with the probe, and [*P*]_0_ is the initial concentration of the probe. By substituting 7 into 3, solving for the reciprocal of *R*_FL_, and rearranging, eq 8 is obtained
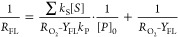
8which describes a linear correlation between the reciprocal of *R*_FL_ and the reciprocal of the probe concentration. By linear interpolation of *R*_FL_^–1^ as a function of [*P*]_0_^–1^, it is possible to obtain the slope [∑ *k*_S_[S]/(*R*_O_2_^–^_Y_FL_k_P_)], the intercept (1/(*R*_O_2_^–^_*Y*_FL_)), and their ratio (eq 9)
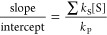
9

Knowing the value of *k*_P_ allows to derive the value of ∑ *k*_S_[S] which is then used in eqs 4 and 7 to obtain the superoxide lifetime, *t*_1/2_, and *F*_O_2_^–^_. *F*_O_2_^–^_ can, in turn, be used to calculate the rate of superoxide formation, *R*_O_2_^–^_, according to eq 3. Finally, using eq 5, the superoxide steady-state concentration, [O_2_^–^]_SS_, can be obtained from ∑ *k*_S_[S] and *R*_O_2_^–^_.

## Results and Discussion

### Spin-Trapping Experiments

**Figure 1:**
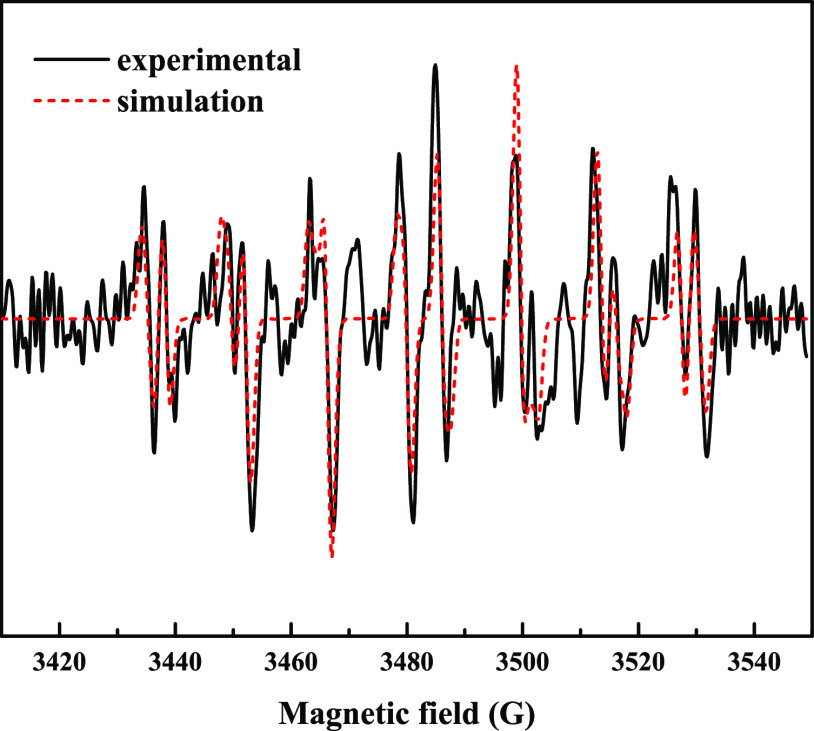
Cw-EPR spectrum of a 9.33 mM solution of DEPMPO plasma treated for 5 min. The red dashed line is the simulated spectrum, obtained as the sum of two species. Species 1: *a*_N_ = 15.24 G, *a*_H1_ = 13.65 G, *a*_H2_ = 0.97 G, and *a*_P_ = 51.11 G; species 2: *a*_N_ = 13.49 G, *a*_H1_ = 14.32 G, *a*_H2_ = 0.41 G, and *a*_P_ = 47.13 G.

The cw-EPR spectrum of a 9.33 mM solution of DEPMPO treated for 5 min in the plasma reactor is reported in Figure 1.

Best-fit simulation of the spectrum revealed that it is the sum of multiple contributions. By comparing the parameters with literature data on DEPMPO, we could identify two main contributions due to the adducts of the spin trap with OH and OOH radicals, respectively.^3^ As reported in the literature,^30^ the simulation was obtained considering only one of the two possible conformers of the OOH adduct, which have very similar parameters and overlapping lines and are thus difficult to distinguish.

### Experiments with PF-1

**Figure 2:**
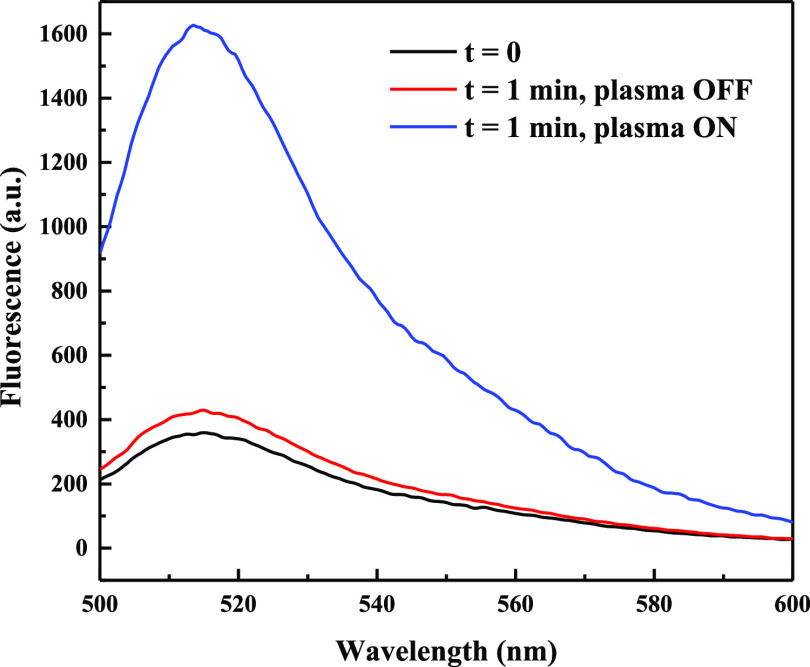
Fluorescence spectra of untreated (black) and treated PF-1 solutions (35 mM, *nominal* concentration in 5 mM phosphate buffer, pH 7) in the reactor flushed with “synthetic air” with plasma OFF (red) and with plasma ON (blue). λ_ex_ = 492 nm, *T* = 25 °C.

Evidence for the production of superoxide in water in our plasma reactor was obtained in experiments in which the fluorescence of PF-1 aqueous solution was measured prior to and after a short plasma treatment. Figure 2 reports the outcome of one such experiment, showing a marked increase of the solution fluorescence following exposure to plasma for 1 min (plasma OFF vs plasma ON). Because it was shown earlier by means of specific tests that most of the reactive species which are also generated by air plasma (H_2_O_2_, ^•^OH, ^1^O_2_, NO, and ONOO^–^) do not give fluorescent products by reaction with PF-1,^24,28^ we can conclude that the increase in fluorescence signal is due to the reaction of PF-1 with superoxide.

We proceeded next to attempt quantitative determinations of the rate of superoxide formation and its steady-state concentration according to the procedure by Anifowose et al.,^24^ which is described in detail in the Experimental Section. This procedure requires the quantitative measurement of both the rate of decay of the probe and the rate of production of FL, which we performed by HPLC/UV–vis and fluorimetric analyses of PF-1 solutions subjected to plasma for different treatment times. To our surprise, however, we could not obtain reproducible and sensible data from these experiments because of the unexpected very low solubility of PF-1 in aqueous solutions. We moved therefore to a more soluble probe, FMSI, the synthesis and properties of which were recently reported in the literature.^27^

### Experiments with FMSI

There are no data in the literature, to the best of our knowledge, showing that the main ROS produced in our system,^25^ HO^•^, O_3_, and probably O and ^1^O_2_, are not interfering in the detection of superoxide by FMSI. We therefore aimed our first experiments at verifying that FL is not formed upon reaction of FMSI with these ROS.

#### Interference Studies

To verify that there are no interferences by the OH radical, we treated a 10 μM aqueous FMSI solution with Fenton reagent (Fe(NH_4_)_2_(SO_4_)_2_ 75 μM + H_2_O_2_ 500 μM, final concentration],^31^ a classical system to generate hydroxyl radicals.^32^ No fluorescence emission was detected in these experiments indicating that, similar to PF-1,^28^ the reaction of FMSI with OH radicals does not produce FL or any other fluorescent product interfering with the detection of superoxide. This is in agreement with the fact that FL is released from the probe upon reaction with strong nucleophiles, including notably superoxide and hydroxide.^12^ Atomic oxygen has no nucleophilic character. Thus, if produced in our system, it would react with the probe via addition to the aromatic system and not via nucleophilic attack on sulfur, likewise hydroxyl radicals.

To test instead for the possible interference by ozone, experiments were carried out using an external ozonizer (Lab-Series ozonizer, A2Z Ozone Inc.). A solution of the probe was placed in the plasma reactor (used simply as reaction vessel with the discharge off) and treated with ozone at a concentration in the gas which was almost 10 times higher than that obtained in the plasma treatment^25^ (the gas flow through the reactor, 0.1 L/min, and the treatment time, 2.5 min, were the same as used in the experiments with plasma). We observed no formation of FL at the fluorimeter. We also verified that failure to detect FL in these experiments with ozone might not be due to its reaction with ozone. Control experiments showed that indeed at the high ozone concentration used in these experiments, some degradation of FL occurs but not to such an extent as to prevent its detection under the conditions cited above. We thus conclude that ozone is not an interfering reactive species.

To test for possible interference of singlet oxygen in the conversion of FMSI into FL, ^1^O_2_ was produced as described in the literature^26^ from NaOCl and H_2_O_2_ in phosphate buffer 200 mM at pH 7 in the presence of FMSI (10 μM). Two different concentrations of NaOCl were investigated, 1 mM and 50 μM, while the concentration of H_2_O_2_ was maintained equal to 100 μM. In both experiments, a fluorescent product was detected but with a distinctly different emission maximum (530 nm) with respect to FL (513 nm). It was verified that the different fluorescence spectrum was not due to a maximum displacement induced by a pH change because the buffered pH remained stable during the reaction. It was thus concluded that singlet oxygen does not produce FL in its reaction with FMSI.

We thus proceeded to test the response of aqueous FMSI to plasma treatment in our reactor. As reported in previous publications,^12^ FMSI can be applied to determine superoxide in solutions only at pH near neutrality (between 6 and 9). This limitation is due to the following reasons: in basic solutions, FMSI produces FL via a different fast route, notably base-induced hydrolysis (eq 10), whereas in acidic solutions, superoxide is protonated (the p*K*_a_ of HOO^•^ is 4.8^33^) and FMSI does not react with HOO^•^ to produce FL (eqs 11 and 12).^12^
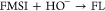
10
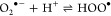
11
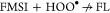
12

It should be noted, however, that, environmental waters have natural buffer systems, which tend to maintain their pH range around neutrality. Thus, the FMSI probe used in this study could be suitably applied to plasma-based treatments of contaminated environmental waters. Thus, all experiments were conducted in a 200 mM phosphate buffer aqueous solution at pH 7.

Another aspect to be considered is that FL produced by plasma treatment of FMSI is expected to be, in turn, degraded due to reaction with ROS, most likely OH radicals. However, it is also expected that at short reaction times, the reaction of FL should be slow because of effective competition for ROS by the FMSI probe, which is present in large excess with respect to FL. These expectations were fulfilled as found in control experiments in which, in place of FMSI, we used phenol, a compound that reacts at a similar rate as FMSI but does not produce any fluorescence upon plasma treatment. Thus, in the presence of phenol (100 μM initial concentration), the rate constant for the decay of FL (1 μM initial concentration) was quite low (0.004 ± 0.006) min^–1^.

#### Yield of FL Formation by Reactions of FMSI with Superoxide

**Figure 3:**
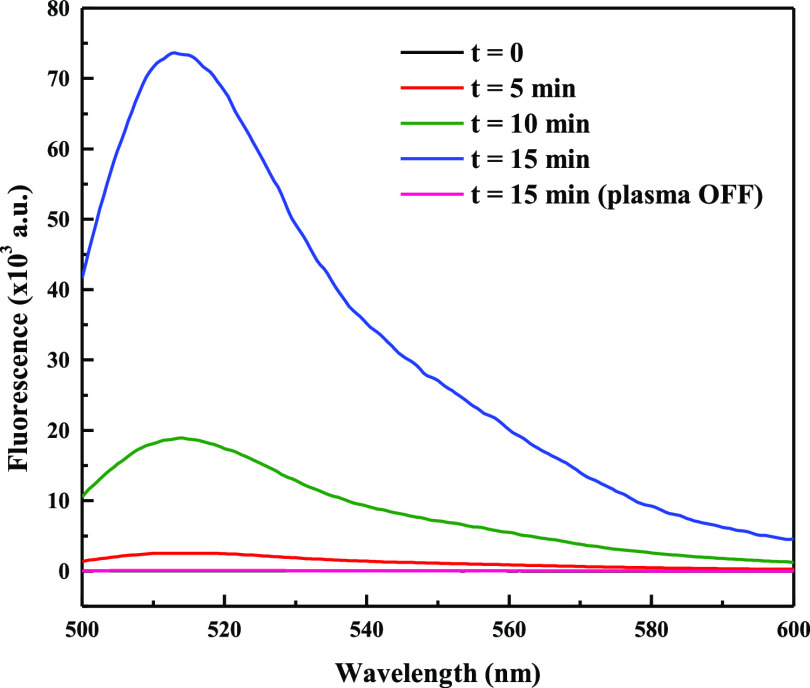
Fluorescence spectra of FMSI aqueous solution (10 μM in 200 mM phosphate buffer) as a function of plasma treatment time. λ_ex_ = 492 nm, *T* = 25 °C.

The fluorescence response of FMSI aqueous solution (10 μM, in 200 mM phosphate buffer) exposed to plasma for different times is shown in Figure 3. It is seen that at the start of the experiment (*t* = 0) and with plasma off (*t* = 15 min plasma OFF), there is no fluorescence. With plasma on, fluorescence is emitted, and the signal intensity increases with increasing plasma treatment time. These results confirm those obtained with PF-1 described in the previous paragraph and show that superoxide is produced in water in response to exposure to air plasma.

**Figure 4:**
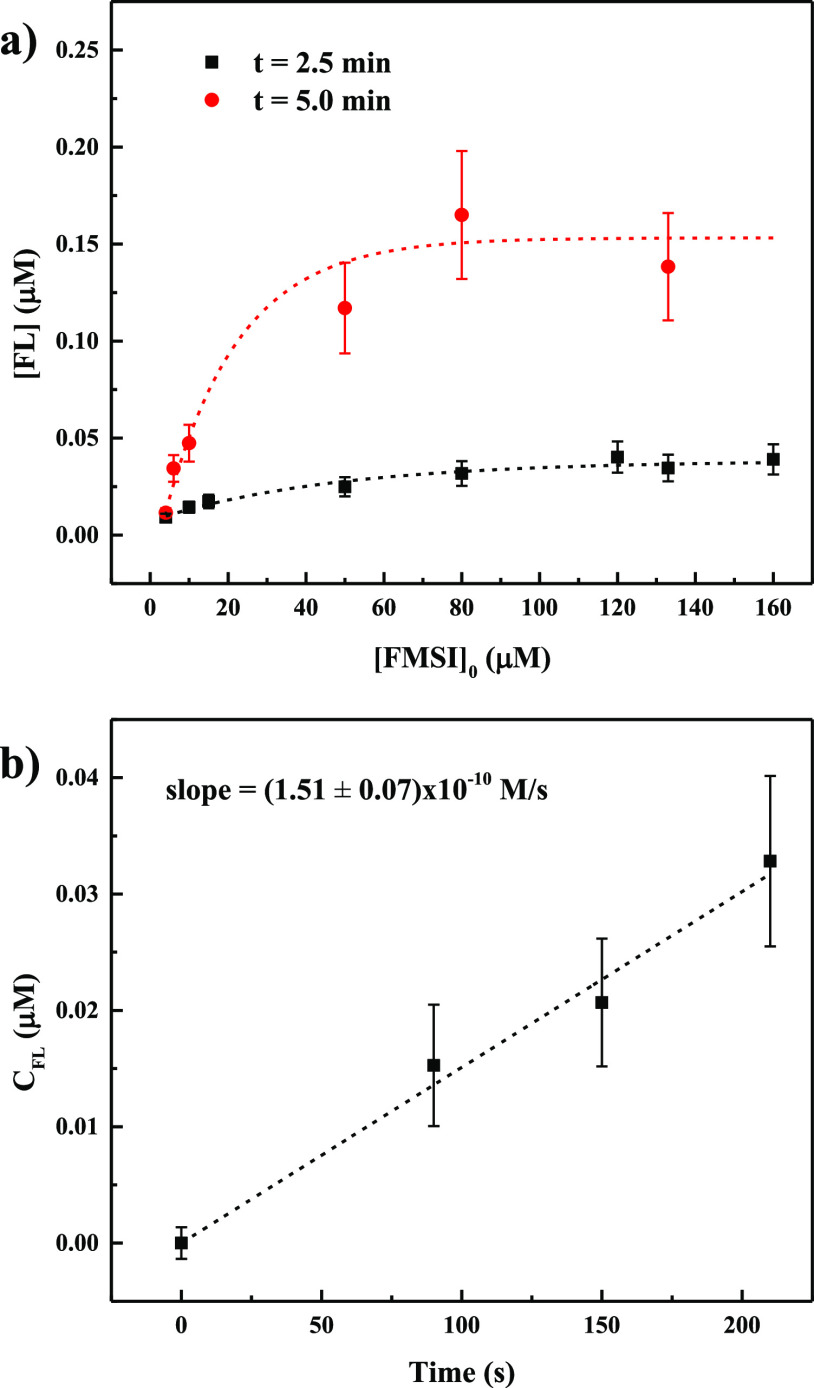
(a) Dependence of FL concentration, produced by plasma treatment, on FMSI starting concentration during 2.5 and 5 min treatments. FL concentration was determined by HPLC/Fluo. The dashed lines are obtained by interpolations of the experimental data with an exponential model ([FL] = [FL]_0_ – *A*e^–*k*[FMSI]_0_^); (b) concentration of FL produced as a function of plasma treatment time during the treatment of a 130 μM FMSI solution in phosphate buffer at pH 7.

Next, we proceeded to the quantitative determination of the rate of formation of superoxide, its lifetime, and its steady-state concentration using the procedure described by Anifowose et al., as detailed in the Experimental Section.^24^ Measurements were thus performed to determine the rate of FMSI decay and that of FL production in experiments run with different FMSI initial concentrations ([FMSI]_0_). The effect of the probe initial concentration on the amount of FL produced is shown in Figure 4a for two different treatment times, 2.5 min (black squares) and 5 min (red circles).

The data show that at a set treatment time, the amount of FL released increases with increasing [FMSI]_0_ until a plateau is reached, indicating that addition of more probe beyond a certain amount is not producing any extra FL. As reasonably expected, the plateau value depends on the treatment time, that is, on the total amount of superoxide produced. Based on these experiments, we chose to work with a FMSI starting concentration of 130 μM in order to be in the plateau region and to use the probe at its full capacity, that is, to capture the maximum possible amount of superoxide generated by plasma. Under these conditions, we determined the yield of FL formation by reaction of FMSI with superoxide (*Y*_FL_) by using eq 6. We obtained the following value: *Y*_FL_ = (0.28 ± 0.08)%.

**Figure 5:**
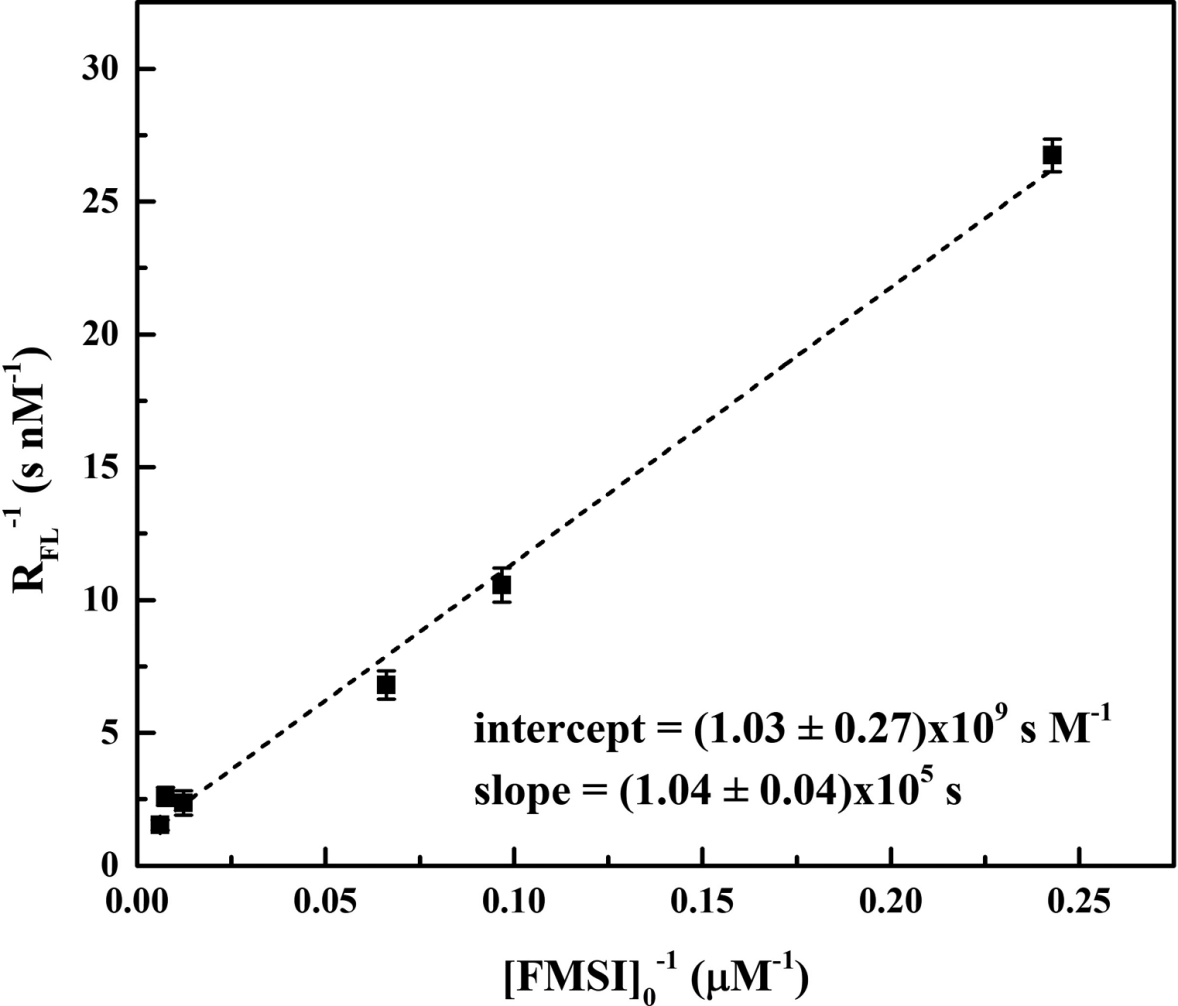
Reciprocal of FL formation rate (1/*R*_FL_) as a function of the reciprocal of [FMSI]_0_. The dashed line is the best linear interpolation of the experimental points. Slope = (1.04 ± 0.04) × 10^5^ s; intercept = (1.03 ± 0.27) × 10^9^ s M^–1^.

Then, following the procedure of Anifowose et al.,^24^ we carried out experiments at various FMSI initial concentrations and determined *R*_FL_ by monitoring, by means of HPLC/Fluo measurements, the amount of FL formed as a function of plasma treatment time. Figure 4b shows the results of the experiment carried out with [FMSI]_0_ = 130 μM. By plotting the reciprocal of *R*_FL_ as a function of the reciprocal of [FMSI]_0_, a reasonably good linear fit of the data is obtained (Figure 5). According to eqs 8 and 9, the ratio between the slope and intercept of this line, equal to (1.00 ± 0.27) × 10^–4^ M, corresponds to the ratio ∑ *k*_S_[S]/*k*_P_, where the term at the denominator refers to the reaction of superoxide with the probe and that at the numerator refers to its reactions with all other scavengers *S* present in the system (excluding the probe).

#### Rate Constant for the Reaction of FMSI with Superoxide

**Figure 6:**
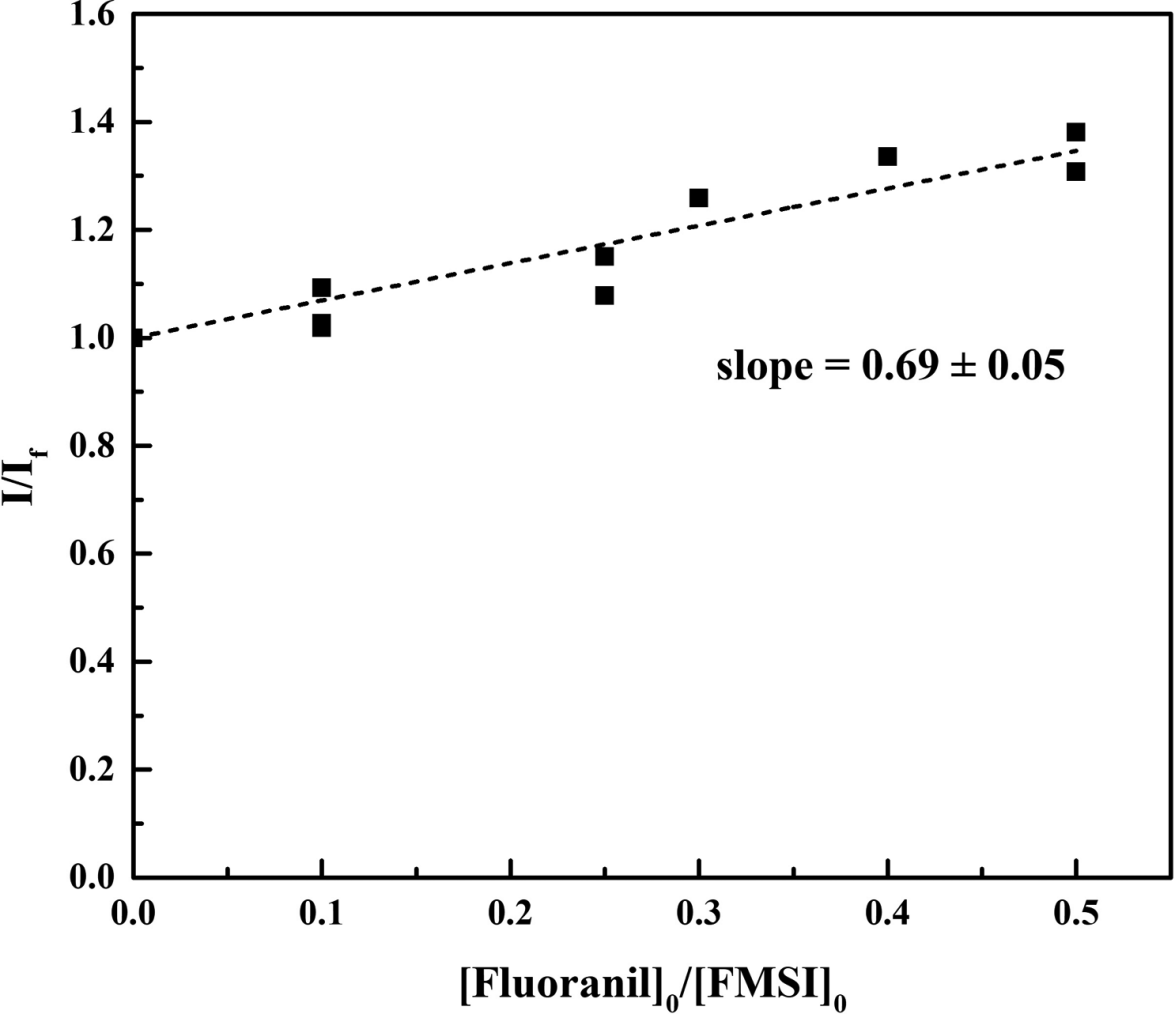
Results of competition kinetics for the reaction of superoxide with FMSI and fluoranil. The experiments were run in 200 mM phosphate buffer at pH 7, with [FMSI]_0_ = 100 μM, [KO_2_] = 1 mM, and various initial concentrations of fluoranil. *I* and *I*_f_ represent the intensity of emitted fluorescence in the absence and in the presence of fluoranil, respectively.

The rate constant for the reaction of FMSI with superoxide, *k*_P_, is not reported in the literature. We therefore determined the rate constant by competitive kinetic analysis using fluoranil as the reference compound and KO_2_ as the source of superoxide.^34^ A small volume of concentrated KO_2_ solution in anhydrous DMSO was added to a cuvette containing FMSI (100 μM) and fluoranil at various initial concentrations (0, 10, 25, 30, 40, and 50 μM) in 200 mM phosphate buffer at pH 7. The reaction was followed by measuring fluorescence as a function of time. Before carrying out these competition kinetic experiments, control experiments showed that fluorescent products do not form when fluoranil is allowed to react with superoxide or mixed in solution with the FMSI probe. The competition of FMSI and fluoranil for superoxide was thus quantified from the decrease of fluorescence, that is, of FL formation when fluoranil was present in the system. The same kinetic treatment described in the literature by Taubert^34^ was applied. So, the ratio between the fluorescence intensity in the absence of fluoranil (*I*) and in its presence (*I*_f_) was plotted as a function of the ratio of the initial concentration of fluoranil and FMSI and fitted by the following equation (Figure 6)
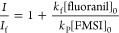
13where *k*_f_ is the kinetic constant of the reaction of fluoranil with superoxide and equal to 2.8·10^8^ M^–1^ s^–1^.^35^ The value of *k*_P_ was thus obtained from the slope of the linear interpolation and is equal to (4.1 ± 0.3) × 10^8^ M^–1^ s^–1^.

#### Superoxide: Consumption by Scavengers, Lifetime, Formation Rate, and Steady-State Concentration

**Figure 7:**
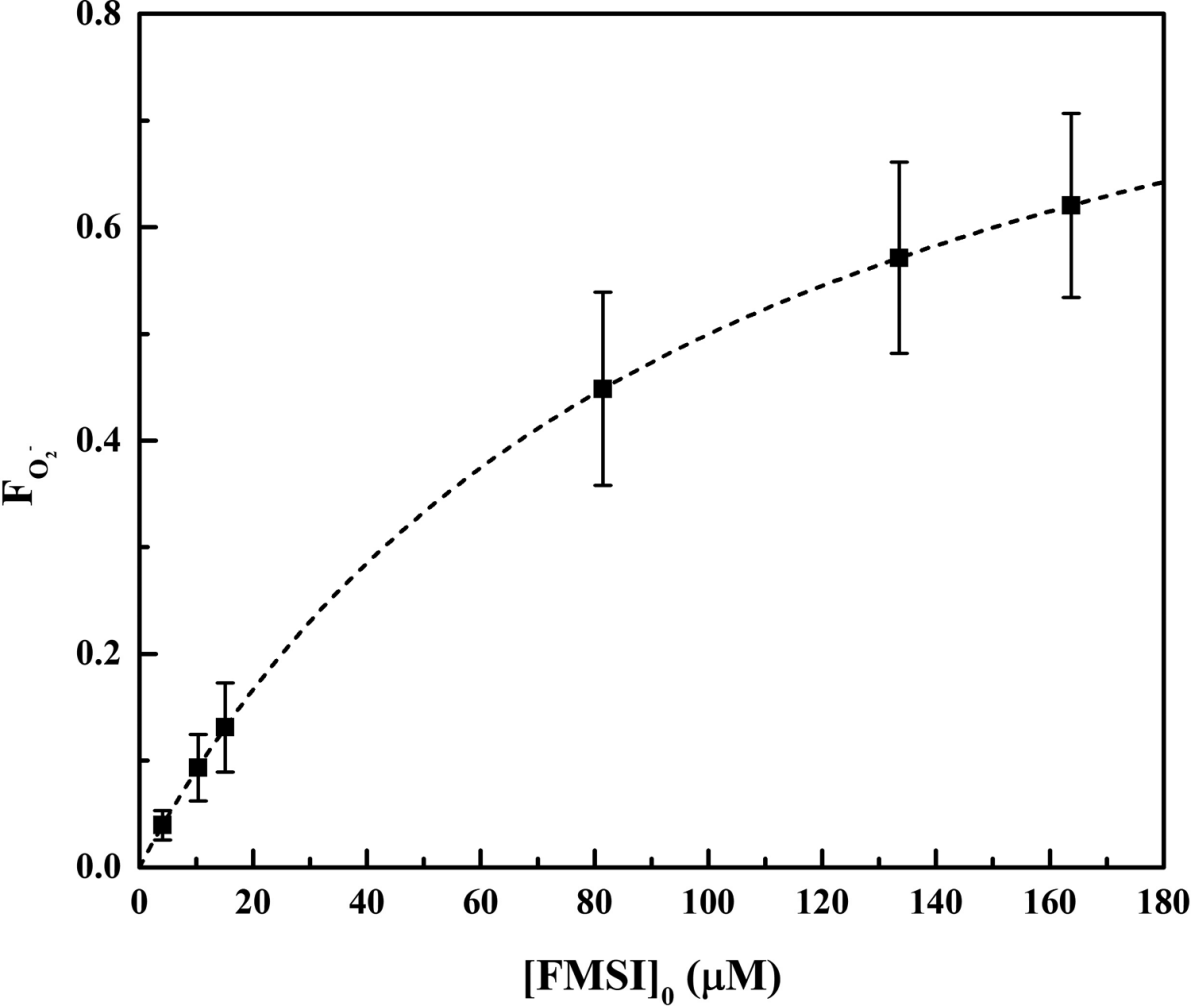
Fraction of plasma-produced superoxide that reacts with FMSI as a function of the probe initial concentration. The dashed line is obtained using eq 7.

Introducing the value of (4.1 ± 0.3) × 10^8^ M^–1^ s^–1^ determined for *k*_P_ in eq 9,^24^ we obtained ∑ *k*_S_[S] = (4.1 ± 1.1) × 10^4^ s^–1^, from which, using eq 4, we calculated that the superoxide lifetime in our system is equal to *t*_1/2_= (1.7 ± 0.5) × 10^–5^ s. Then, using eq 7, the fraction of superoxide captured by the probe (*F*_O_2_^–^_) was calculated to be 0.57 ± 0.09 under the experimental conditions adopted ([FMSI]_0_ = 130 μM). Clearly, *F*_O_2_^–^_ depends on the probe concentration (Figure 7) and tends to 1 when [FMSI]_0_ ≫ ∑ *k*_S_[S] (eq 7). Finally, using eqs 3 and 5 we calculated, respectively, the rate of superoxide formation, *R*_O_2_^–^_, and its steady-state concentration, [O_2_^–^]_SS_. We obtained the following values: *R*_O_2_^–^_ = (2.7 ± 1.5) × 10^–7^ M s^–1^ and [O_2_^–^]_SS_ = (7 ± 4) × 10^–12^ M. Table 1 shows all relevant data obtained in this study.

**Table 1:** Summary of Quantitative Data Determined in This Work on Superoxide Generated in Air Non-thermal Plasma Reactor^a^

parameter	value
∑ *k*_S_[S] (s^–1^)	(4.1 ± 1.1) × 10^4^
*t*_1/2_ (s)	(1.7 ± 0.5) × 10^–5^
*R*_O_2_^–^_ (M·s^–1^)	(2.7 ± 1.5) × 10^–7^
[O_2_^–^]_SS_ (M)	(7 ± 4) × 10^–12^

a Scavenging rate of superoxide (∑ *k*_S_[S]), half-life time of superoxide (*t*_1/2_), rate of formation of superoxide (*R*_O_2_^–^_), and steady-state concentration of superoxide in solution ([O_2_^–^]_SS_).

## Conclusions

As mentioned in the introduction, there has been no previous attempt, to the best of our knowledge, to apply fluorescence-generating probes to determine superoxide production in water exposed to air non-thermal plasma. Moreover, only very few papers reported quantitative data of superoxide concentration in related systems, estimated using alternative approaches, with which it would have been interesting to compare our results. This is not possible, however, because these data are not superoxide concentrations but rather the amounts of superoxide trapped by the chemical probe (spin trap or other) over the duration of the plasma treatment.

We can instead compare our results with those reported by Anifowose et al. for the rate of formation and steady-state concentration of superoxide in waters exposed to natural sunlight.^24^ The rate of superoxide formation, *R*_O_2_^–^_, in our plasma reactor is *ca.* 3 × 10^–7^ M·s^–1^, which is about 2 orders of magnitude larger than that photoinduced by solar irradiation, 6 × 10^–9^ M·s^–1^.^24^ This observation is certainly not surprising and might lead to the expectation that a higher steady-state concentration of superoxide, [O_2_^–•^]_SS_, might thus be achieved in plasma-treated water. This anticipation is, however, not fulfilled because the value determined in our system [(7 ± 4) × 10^–12^ M] is slightly lower than that found in seawaters (1.3 × 10^–11^ M).^24^ A rationale for these observations is found in the considerably larger value of ∑ *k*_S_[S] determined in plasma-treated water than in sun-irradiated water, (4.1 ± 1.1) × 10^4^ versus 5.5 × 10^2^ s^–1^, respectively, which results in a shorter lifetime. So, although the rate of superoxide formation is much higher in water treated by plasma than by solar irradiation, the steady-state concentration of this reactive species is lower due to the occurrence of efficient destruction reactions. Specifically, one should consider the reactions with OH radicals, with ozone, and with NO^•^ (eqs 14–16), species which are all formed in plasma-treated water.^11,17,36^
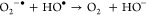
14
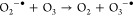
15
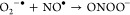
16

It should be noted that although reaction 14 consumes OH radicals, the products of reactions 15 and 16, ozone radical anion and peroxynitrite, respectively, react to regenerate OH radicals as shown in eqs 17, 18 and 19, 20, respectively^11,36^
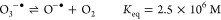
17
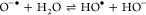
18
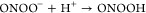
19
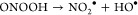
20

Moreover, if one considers generic water treated by plasma, the possible presence of transition metals (such as Fe^2+^ and Cu^+^) originating from the electrodes has also to be taken into consideration because they catalyze the reaction of superoxide with hydrogen peroxide through the Haber–Weiss reaction 21.^17^
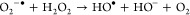
21

Therefore, superoxide in plasma-treated water is a source of OH radicals, which are among the strongest oxidizing species in nature. In previous studies on water treatment with the plasma reactor used in this investigation, we had indeed concluded that OH radicals were the main reactive species initiating the degradative oxidation process of organic pollutants.^25,37^
